# Physiological Responses and Transcriptome Analysis of ‘Hanfu’ Apple Plants Under PEG-Simulated Drought Stress

**DOI:** 10.3390/biology15201861

**Published:** 2026-10-16

**Authors:** Xingzhu Chen, Tongyu Zhou, Deguo Lyu, Lijie Li, Sijun Qin

**Affiliations:** College of Horticulture, Shenyang Agricultural University, Shenyang 110866, China; 2024220352@stu.syau.edu.cn (X.C.); zhoutongyu0526@163.com (T.Z.); lvdeguo@syau.edu.cn (D.L.)

**Keywords:** ‘Hanfu’ apple, drought stress, photosynthetic pigments, antioxidant system, plant hormone signal transduction

## Abstract

Drought stress constitutes a major constraint on apple growth, development, and yield. Although drought responses have been extensively studied in apple, the early physiological and transcriptomic responses of ‘Hanfu’, a cultivar widely cultivated in the cool regions of northern China and reported to possess drought resistance, remain insufficiently characterized. Therefore, this study integrated physiological measurements and transcriptome analysis to characterize the short-term drought responses of ‘Hanfu’ apple. One-year-old grafted ‘Hanfu’ apple seedlings were grown hydroponically and then treated with 5%, 10%, and 15% PEG-6000 for 6, 12, 24, and 48 h to impose PEG-induced osmotic stress as a simulation of drought-related water deficit. Leaf water potential, photosynthetic pigments, superoxide anion (O_2_·^−^), hydrogen peroxide (H_2_O_2_), malondialdehyde (MDA), and antioxidant enzyme activities were measured. RNA sequencing identified differentially expressed genes (DEGs), enriched pathways, and drought-responsive transcription factor families. The results showed that PEG-simulated drought stress aggravated leaf water deficit, reduced photosynthetic pigments, and downregulated photosynthetic-related genes expression (*CAB*, *CP24*, *PSA*, and *PNSL2*). ROS and MDA levels were increased, activities of SOD and POD were enhanced, and antioxidant-related genes (*PPO*, *APX*, *PK1*, and *PER*) were upregulated. Genes involved in plant hormone signal transduction showed marked differential expression, particularly those associated with the IAA and ABA signaling pathways. Related genes such as *ARF5*, *GH3.1*, *SAUR32*, *ARG7*, *PP2C*, *ABI*, and *HAB1* could play important roles in responding to PEG-simulated drought stress. Also, MYB, WRKY, bZIP, AP2, bHLH, Dof, NAC, and ARF may be the major transcription factor families involved. Overall, ‘Hanfu’ apple leaves may coordinate photosynthesis, antioxidant defense, IAA and ABA signaling, and transcriptional regulation to respond to drought stress. These findings provide a physiological and molecular basis for further functional validation of drought-responsive genes and may contribute to future breeding for drought resistance and drought management in apple.

## Simple Summary

Drought is a major environmental constraint on apple growth and yield in China, particularly in arid and semi-arid apple-producing regions. However, the integrated physiological and transcriptomic responses of ‘Hanfu’ apple to short-term drought stress remain insufficiently characterized. In this study, we assessed drought-induced alterations in leaf physiological traits and transcriptional responses of ‘Hanfu’ apple seedlings. PEG-simulated drought stress reduced leaf water potential and photosynthetic pigment contents, while increasing oxidative damage and enhancing antioxidant enzyme activities. Transcriptome analysis showed that PEG-simulated drought stress affected photosynthesis, antioxidant defense, and plant hormone signal transduction, particularly the auxin and abscisic acid signaling pathways. These findings deepen our understanding of the drought response of ‘Hanfu’ apple leaves and offer candidate genes for subsequent studies of drought tolerance in apple.

## 1. Introduction

Against the backdrop of global climate change, drought has become one of the major abiotic stresses limiting apple growth and development and threatening the sustainable development of the apple industry. Currently, drylands cover approximately 42% of the global land area, and drought events are becoming more frequent and severe in many regions under climate change [1,2]. In apple, drought stress disrupts plant water status, leading to a progressive decline in leaf water potential as stress intensifies. Meanwhile, chlorophyll and carotenoid contents decrease, which adversely affects leaf photosynthetic function [3,4]. Studies on ‘Hanfu’ apple (*Malus domestica* Borkh.) have shown that drought stress accelerates chlorophyll degradation, suppresses photosynthesis and photosystem II (PSII) function, and promotes H_2_O_2_ accumulation [5]. Drought stress also induces the accumulation of O_2_·^−^ and H_2_O_2_ in apple leaves, accompanied by an increase in malondialdehyde (MDA) content, indicating enhanced membrane lipid peroxidation and cell membrane damage. Under drought-induced oxidative stress, superoxide dismutase (SOD) and peroxidase (POD) function as key components of the enzymatic defense system and help limit excess ROS accumulation. Their activities are dynamically regulated as drought stress progresses, thereby contributing to the alleviation of oxidative damage [6,7].

The effects of drought on plants are reflected not only at the levels of growth, physiology, and metabolism, but also in complex molecular regulatory processes. Studies have shown that drought stress can activate ABA-dependent and ABA-independent signal transduction pathways. In the core ABA signaling pathway, ABA is perceived by PYR/PYL/RCAR receptors, which inhibit PP2C phosphatases and thereby promote the activation of SnRK2 kinases to regulate downstream stress responses [8]. In addition to the ABA signaling pathway, the DREB/CBF-mediated ABA-independent signal transduction pathway also participates in the regulation of plant drought responses [9]. In apple, drought stress can likewise alter the expression of related genes. For example, under drought conditions, genes such as *MdNRT2.4* and *MpNPR1-2* are upregulated in apple roots [10]. In addition, the transcription factors MdMYB88 and MdMYB124 regulate root xylem development by directly targeting the promoters of *MdVND6* and *MdMYB46* and inducing their expression, and also modulate cellulose and lignin deposition in root cell walls, thereby contributing to drought adaptation in apple [11]. Drought stress also induces the expression of *MdERF38*. The MdERF38 protein interacts with MdMYB1 and facilitates the binding of MdMYB1 to its target genes, thereby promoting drought stress-induced anthocyanin biosynthesis [12]. Moreover, drought-induced expression of the ABA receptor gene *MdPYL9* can increase relative water content and the photosynthetic rate while reducing ROS accumulation [13]. At the transcriptional regulatory level, stress-related transcription factors, such as MYB, bHLH, bZIP, WRKY, and NAC, are also differentially expressed under drought conditions and jointly participate in the molecular regulation of plant responses to drought stress [14].

In China, an estimated 1.0 million ha of apple orchards are located in arid or semi-arid regions, where water deficits can inhibit the growth and leaf photosynthesis of apple plants, thereby affecting fruit yield and quality [15,16]. Previous studies on apple drought responses have mainly focused on physiological traits, including leaf water status, photosynthetic performance, ROS accumulation, membrane lipid peroxidation, and antioxidant enzyme activities, as well as molecular responses involving ABA signaling, individual drought-responsive genes, and stress-related transcription factors. However, the integrated physiological and transcriptomic responses during the early stage of drought stress remain insufficiently characterized, particularly in ‘Hanfu’ apple. Polyethylene glycol 6000 (PEG-6000) is a non-toxic, non-ionic polymer with an average molecular weight of approximately 6000. PEG-6000 is widely used to impose controlled osmotic stress by reducing external water potential and thereby simulate drought-related water deficit [17]. ‘Hanfu’ is an apple cultivar widely cultivated in the cool regions of northern China and has been reported to possess cold and drought resistance [18]. However, its early physiological and transcriptomic responses to drought stress remain poorly understood. In this study, ‘Hanfu’ apple seedlings were used as the experimental material, and PEG-6000 was applied to simulate drought stress. We hypothesized that PEG-simulated drought stress would induce coordinated physiological responses in apple leaves in a concentration- and time-dependent manner, and induce early transcriptional reprogramming in ‘Hanfu’ apple leaves. To test this hypothesis, we analyzed the characterize changes in leaf water potential, photosynthetic pigments, ROS, MDA, and antioxidant enzyme activities under different PEG-6000 concentrations and treatment durations; and the early transcriptomic responses of ‘Hanfu’ apple leaves to PEG-simulated drought stress at 6 and 24 h. This work aims to identify candidate drought-responsive genes for future functional validation and provide a theoretical basis for optimizing apple cultivation practices in arid regions.

## 2. Materials and Methods

### 2.1. Plant Materials and Treatments

One-year-old grafted ‘Hanfu’ apple seedlings provided by Shenyang Agricultural University (Shenyang, China) were used as the experimental materials. ‘Hanfu’ was grafted onto GM256 as the interstock, with *Malus hupehensis* Rehd. used as the base rootstock. Before PEG-6000 treatment, the grafted seedlings were grown individually in plastic pots (30 cm × 30 cm) containing a mixture of orchard soil collected from the apple orchard of Shenyang Agricultural University, sand, and substrate at a ratio of 4:4:2. The potting soil contained 15.25 g·kg^−1^ organic matter, 78.09 mg·kg^−1^ alkali-hydrolyzable nitrogen, 25.62 mg·kg^−1^ available phosphorus, and 158.21 mg·kg^−1^ available potassium, with a pH of 6.5. The seedlings were maintained under a rain shelter with routine management before treatment. The experiment was conducted in August in Shenyang. Under the local natural seasonal conditions, the mean air temperature was approximately 23.8 °C, the mean relative humidity was approximately 79%, and the natural photoperiod was approximately 13.8 h light/10.2 h dark.

Seedlings exhibiting uniform growth and approximately 80 cm in height were selected, after which the soil was carefully removed from the roots. The roots were rinsed three times with tap water and once with distilled water and then transferred to Hoagland nutrient solution for a 7-d acclimation period. Following acclimation, the seedlings were transferred to opaque plastic buckets with a nominal capacity of 20 L (approximately 35 cm in upper diameter, 26.5 cm in bottom diameter, and 31 cm in height) for PEG-6000 treatment. The PEG-6000 treatment was also conducted under the same rain-shelter conditions described above. For each treatment at each sampling time, three independent buckets were used as biological replicates, with two seedlings placed in each bucket, resulting in six seedlings per treatment and sampling time. Each bucket contained approximately 15 L of treatment solution. The treatment solutions were renewed once daily throughout the experimental period using freshly prepared solutions with the corresponding PEG-6000 concentrations to maintain consistent treatment conditions.

PEG-6000 (Shanghai Macklin Biochemical Technology Co., Ltd., Shanghai, China) solutions were prepared at concentrations of 0%, 5%, 10%, and 15% (*w*/*v*) at room temperature (approximately 25 °C). According to the relationship between PEG-6000 concentration and osmotic potential described by Michel and Kaufmann [18], the estimated PEG-induced osmotic potentials corresponding to 0%, 5%, 10%, and 15% PEG-6000 were approximately 0, −0.05, −0.15, and −0.30 MPa, respectively. The treatments were designated as CK, T1, T2, and T3, respectively. The CK seedlings were subjected to the same transfer procedure, root-immersion conditions, aeration, treatment duration, environmental conditions, and sampling procedures as the PEG-treated seedlings, except that no PEG-6000 was added to the CK treatment.

Because the roots were completely immersed during treatment, continuous aeration was supplied throughout the experimental period to maintain oxygen availability in the root zone and minimize the potential for hypoxia. Aeration was provided using SB-848 dual-outlet air pumps (Zhongshan SOBO Electric Appliance Co., Ltd., Zhongshan, China; 12 W, 220–240 V), with a manufacturer-rated airflow of 4.5 L·min^−1^ per outlet. The CK seedlings were subjected to the same transfer procedure, root-immersion conditions, aeration, treatment duration, environmental conditions, and sampling procedures as the PEG-treated seedlings, except that no PEG-6000 was added to the CK treatment.

Samples were collected at 6, 12, 24, and 48 h after treatment. For each treatment at each sampling time, six seedlings were used, resulting in a total of 96 seedlings. The fourth to sixth leaves from the shoot apex were collected by destructive sampling, immediately frozen in liquid nitrogen, and stored at −80 °C until further analysis.

### 2.2. Measurement of Leaf Water Potential

Leaf water potential was determined using a Psypro water potential system (Wescor, Inc., Logan, UT, USA). Measurements were conducted at 10:00 on sunny days using the fourth to sixth leaves from the shoot apex. Leaves of comparable size and from the same relative position were selected for all treatments [19].

### 2.3. Measurement of Superoxide Anion, Hydrogen Peroxide, and Malondialdehyde Contents

Superoxide anion (O_2_·^−^) and hydrogen peroxide (H_2_O_2_) levels were determined following the method described by Zhou et al. Absorbance was recorded at 530 and 410 nm, respectively, using a TU-1900 UV–visible spectrophotometer (Beijing Purkinje General Instrument Co., Ltd., Beijing, China). The determination of malondialdehyde (MDA) content was carried out according to the procedure reported by Zhou et al. [20].

### 2.4. Measurement of Photosynthetic Pigments

The contents of chlorophyll a, chlorophyll b, carotenoids, and total chlorophyll were determined according to the method of Tang et al. [21]. Fresh leaf samples (0.2 g) were immersed in 80% acetone (Shanghai Macklin Biochemical Technology Co., Ltd., Shanghai, China) and extracted in the dark for 24 h. The absorbance of the extract was measured at 663, 645, and 470 nm using a spectrophotometer. The pigment contents were calculated using the following equations:

Chl a (mg·g^−1^ FW) = (12.7*A*_663_ − 2.69*A*_645_) × *V*/(1000 *W*)

Chl b (mg·g^−1^ FW) = (22.9*A*_645_ − 4.68*A*_663_) × *V*/(1000 *W*)

Total Chl (mg·g^−1^ FW) = (20.20*A*_645_ + 8.02*A*_663_) × *V*/(1000 *W*)

Carotenoids (mg·g^−1^ FW) = [(1000*A*_470_ − 3.27*C*_a_ − 104*C*_β_)/229] × *V*/(1000 *W*),

where *A*_663_, *A*_645_, and *A*_470_ represent the absorbance values at 663, 645, and 470 nm, respectively; *V* represents the final volume of the extract (mL); *W* represents the fresh weight of the sample (g); and *C*_a_ and *C*_β_ represent the concentrations of chlorophyll a and chlorophyll b, respectively.

### 2.5. Measurement of Peroxidase and Superoxide Dismutase Activities

Peroxidase (POD) activity was assayed following the procedure described by He et al. [17,22]. Superoxide dismutase (SOD) activity was quantified with an assay kit obtained from Shanghai Enzyme-linked Biotechnology Co., Ltd. (Shanghai, China), and the procedure was carried out as specified by the supplier.

### 2.6. RNA Extraction, cDNA Library Construction, and Transcriptome Data Analysis

Total RNA was isolated from apple leaves using an optimized CTAB method [23]. RNA purity was assessed based on the OD260/280 ratio (1.9–2.2), and RNA integrity was evaluated prior to library construction. Only RNA samples with RIN values ≥ 7.0 that met the quality requirements for plant whole-transcriptome sequencing were used for library preparation. RNA samples were subsequently delivered to Novogene Bioinformatics Technology Co., Ltd. (Beijing, China). cDNA libraries were prepared there, followed by sequencing. Three independent biological replicates were included for each treatment, with each biological replicate corresponding to one independently treated bucket containing two seedlings. The cDNA libraries were prepared for high-throughput sequencing. The resulting libraries were then sequenced on an Illumina HiSeq 2500 platform (Illumina, Inc., San Diego, CA, USA).

Following base calling with CASAVA (version 1.8), the sequencing data were converted into raw reads. Adapter sequences, reads containing ambiguous bases, and low-quality reads were filtered out to generate clean reads. The resulting clean reads were mapped to the *Malus × domestica* GDDH13 Whole Genome v1.1 reference genome using HISAT2 (version 2.0.5) [24]. Read counts for individual genes were obtained using featureCounts (version 1.5.0-p3). Gene expression abundance was quantified as FPKM (fragments per kilobase of exon model per million mapped fragments), thereby normalizing for differences in sequencing depth and gene length.

### 2.7. Identification and Enrichment Analysis of DEGs

DESeq2 (version 1.16.1) was used to compare gene expression between the treatment and control groups based on the normalized expression data. Adjusted *p*-values were then calculated to assess statistical significance [25]. Genes satisfying the thresholds of |log2FC| ≥ 1 and FDR < 0.05 were defined as differentially expressed genes (DEGs) [26]. The identified DEGs were subjected to Gene Ontology (GO) enrichment analysis to determine significantly enriched biological process (BP), cellular component (CC), and molecular function (MF). Kyoto Encyclopedia of Genes and Genomes (KEGG) enrichment analysis was subsequently conducted to identify the metabolic and signal transduction pathways significantly associated with the DEGs.

### 2.8. Quantitative Real-Time PCR (qRT-PCR) Validation

RNA samples were converted to cDNA using a kit supplied by Vazyme Biotech Co., Ltd. (Nanjing, China), according to the supplier’s instructions. Gene-specific primers were designed using the online Primer3.0 tool, and their sequences are provided in Supplementary Table S1.

Quantitative real-time PCR (qRT-PCR) was conducted using SYBR^®^ Green Real-time PCR Master Mix (ComWin Biotech Co., Ltd., Beijing, China), with the reaction mixture prepared as recommended by the manufacturer. The amplification conditions consisted of an initial denaturation at 95 °C for 30 s, followed by 40 cycles at 95 °C for 5 s and 60 °C for 30 s. Reactions were carried out in 384-well plates, using *Apple-Actin* as the reference gene [27]. The 2^−ΔΔCT^ method was used to calculate relative gene expression [28]. Three technical replicates were performed for each biological replicate.

### 2.9. Transcription Factor (TF) Analysis

The identified DEGs were compared against the Plant Transcription Factor Database (PlantTFDB v5.0; PlantTFDB—Plant Transcription Factor Database @ CBI, PKU) to identify differentially expressed transcription factors, which were subsequently classified according to their functions or characteristics.

### 2.10. Data Analysis

Statistical analyses were performed using IBM SPSS Statistics 31.0.1 (IBM Corp., Armonk, NY, USA). At each sampling time point, differences among the CK, T1, T2, and T3 treatments were analyzed separately using one-way analysis of variance (ANOVA), followed by Tukey’s honestly significant difference (HSD) test for multiple comparisons. Statistical significance was accepted at *p* < 0.05. Figures were generated using Origin 2025 (version 10.2; OriginLab Corporation, Northampton, MA, USA).

## 3. Results

### 3.1. Changes in Leaf Water Potential Under Different PEG-6000 Treatments

**Figure 1:**
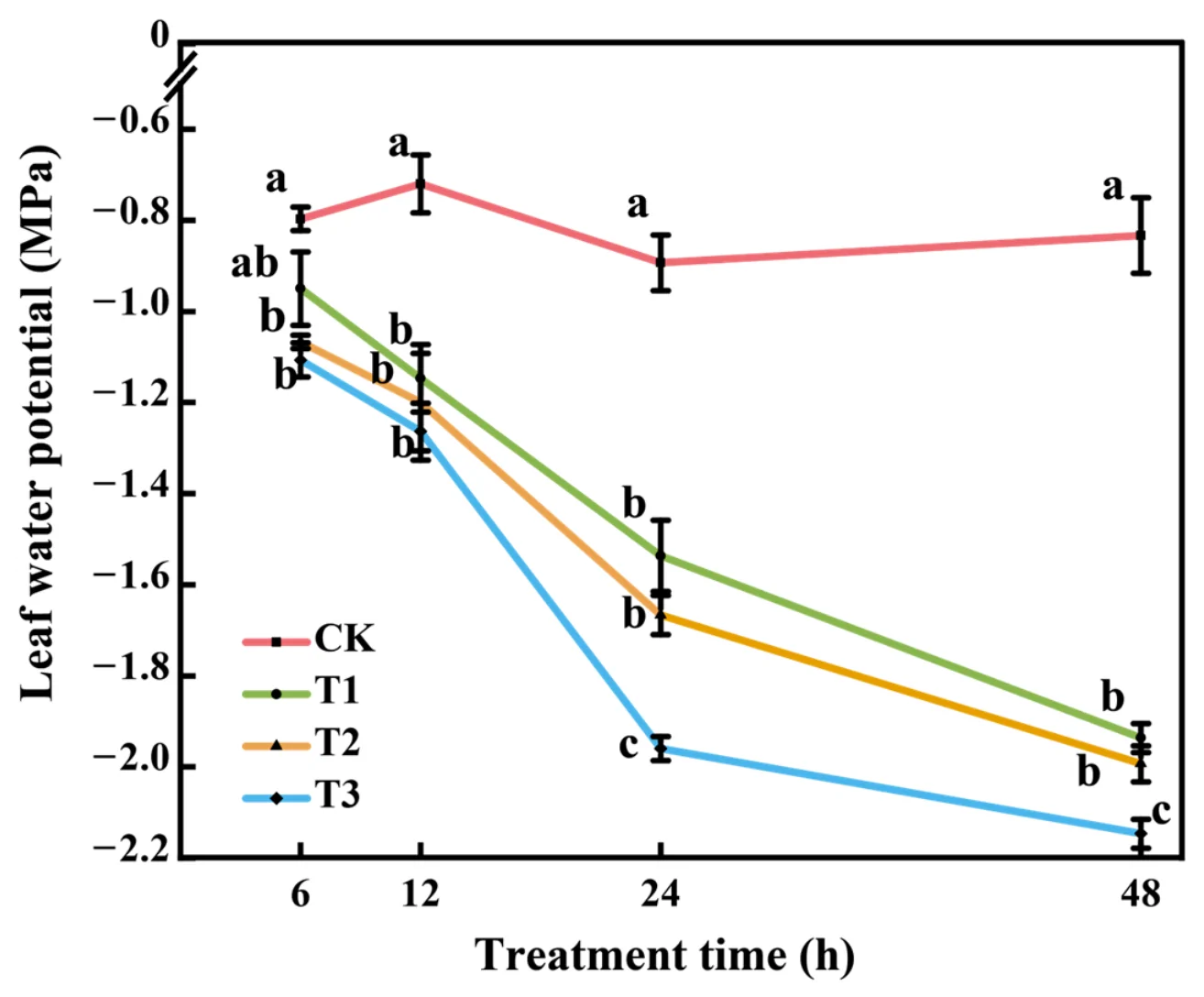
Effect of PEG treatment on water potential of apple leaves. Note: CK represents the 0% PEG-6000 treatment, whereas T1, T2, and T3 represent the 5%, 10%, and 15% PEG-6000 treatments, respectively. Data are reported as mean ± standard error (SE). At each sampling time, different letters indicate significant differences among treatments according to Tukey’s honestly significant difference (HSD) test (*p* < 0.05).

Compared with CK, all PEG-6000 treatments significantly decreased leaf water potential (Figure 1). With increasing treatment duration, leaf water potential showed no significant change in CK, whereas it exhibited a decreasing trend in the T1, T2, and T3 treatments. At 24 h, leaf water potential in the T3 treatment was significantly lower than that in the T1 and T2 treatments (*p* = 0.0028 and *p* = 0.0231) and remained the lowest among all treatments at all subsequent sampling time points.

### 3.2. Changes in Leaf Photosynthetic Pigment Contents Under Different PEG-6000 Treatments

**Figure 2:**
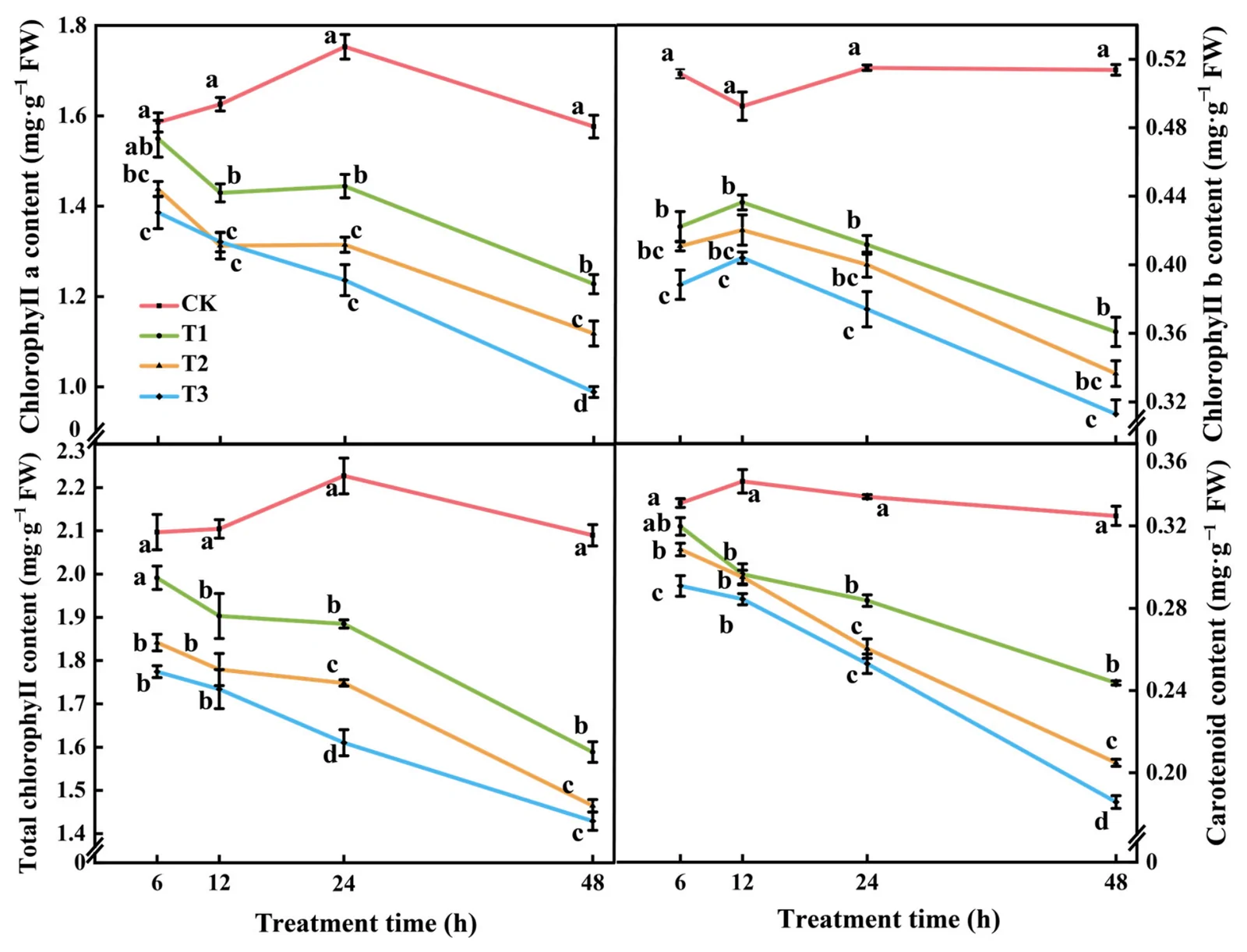
Effects of PEG treatment on the contents of photosynthetic pigments in apple leaves. Note: CK represents the 0% PEG-6000 treatment, whereas T1, T2, and T3 represent the 5%, 10%, and 15% PEG-6000 treatments, respectively. Data are presented as mean ± standard error (SE). At each sampling time, different letters indicate significant differences among treatments according to Tukey’s honestly significant difference (HSD) test (*p* < 0.05).

Chlorophyll is a major photosynthetic pigment involved in plant photosynthesis, and changes in its content can reflect alterations in photosynthetic function. Compared with CK, treatments with different concentrations of PEG-6000 significantly reduced the contents of chlorophyll a, total chlorophyll, and carotenoids in the leaves (Figure 2). With increasing treatment duration, no significant changes were observed in CK, whereas the contents of chlorophyll a, total chlorophyll, and carotenoids in the T1, T2, and T3 treatments showed decreasing trends. At 48 h, the T3 treatment exhibited the lowest contents of chlorophyll a, total chlorophyll, and carotenoids, which were 37.3%, 31.6%, and 42.8% lower than those in CK, respectively; chlorophyll a content was also significantly lower in T3 than in T2 (*p* = 0.0152).

Under PEG-6000 treatment, the chlorophyll b content initially increased and then decreased. At 6 and 12 h, the chlorophyll b content in the T1, T2, and T3 treatments showed a transient increase and then gradually decreased. By 48 h, the chlorophyll b content in all PEG-6000 treatment groups had decreased significantly relative to CK.

### 3.3. Changes in Leaf Membrane Lipid Peroxidation and Reactive Oxygen Species Under Different PEG-6000 Treatments

**Figure 3:**
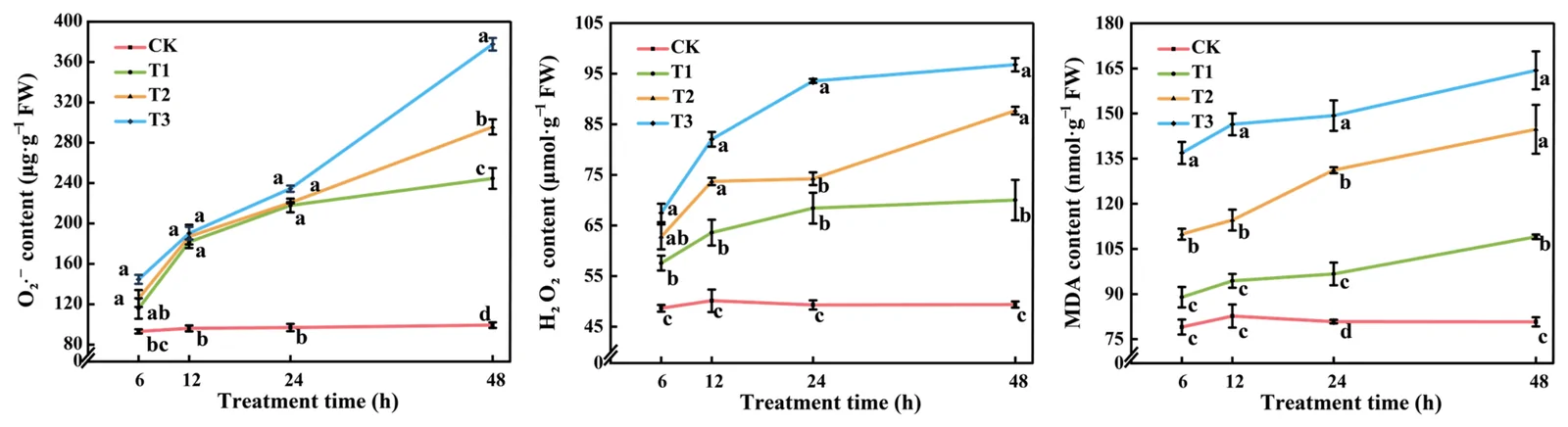
Effects of PEG treatment on O_2_·^−^, H_2_O_2_ and MDA contents in apple leaves. Note: CK represents the 0% PEG-6000 treatment, whereas T1, T2, and T3 represent the 5%, 10%, and 15% PEG-6000 treatments, respectively. Data are presented as mean ± standard error (SE). At each sampling time, different letters indicate significant differences among treatments according to Tukey’s honestly significant difference (HSD) test (*p* < 0.05).

Relative to CK, O_2_·^−^, H_2_O_2_, and MDA levels in leaves under different concentrations of PEG-6000 treatment increased with prolonged stress duration (Figure 3). With increasing treatment duration, no significant changes were observed in CK, whereas the contents of O_2_·^−^, H_2_O_2_, and MDA increased significantly in the T1, T2, and T3 treatments. At 48 h, the T3 treatment showed the highest contents of O_2_·^−^, H_2_O_2_, and MDA, which were 280.2%, 96.0%, and 103.5% higher than those in CK, respectively.

### 3.4. Changes in Leaf Antioxidant Enzyme Activities Under Different PEG-6000 Treatments

**Figure 4:**
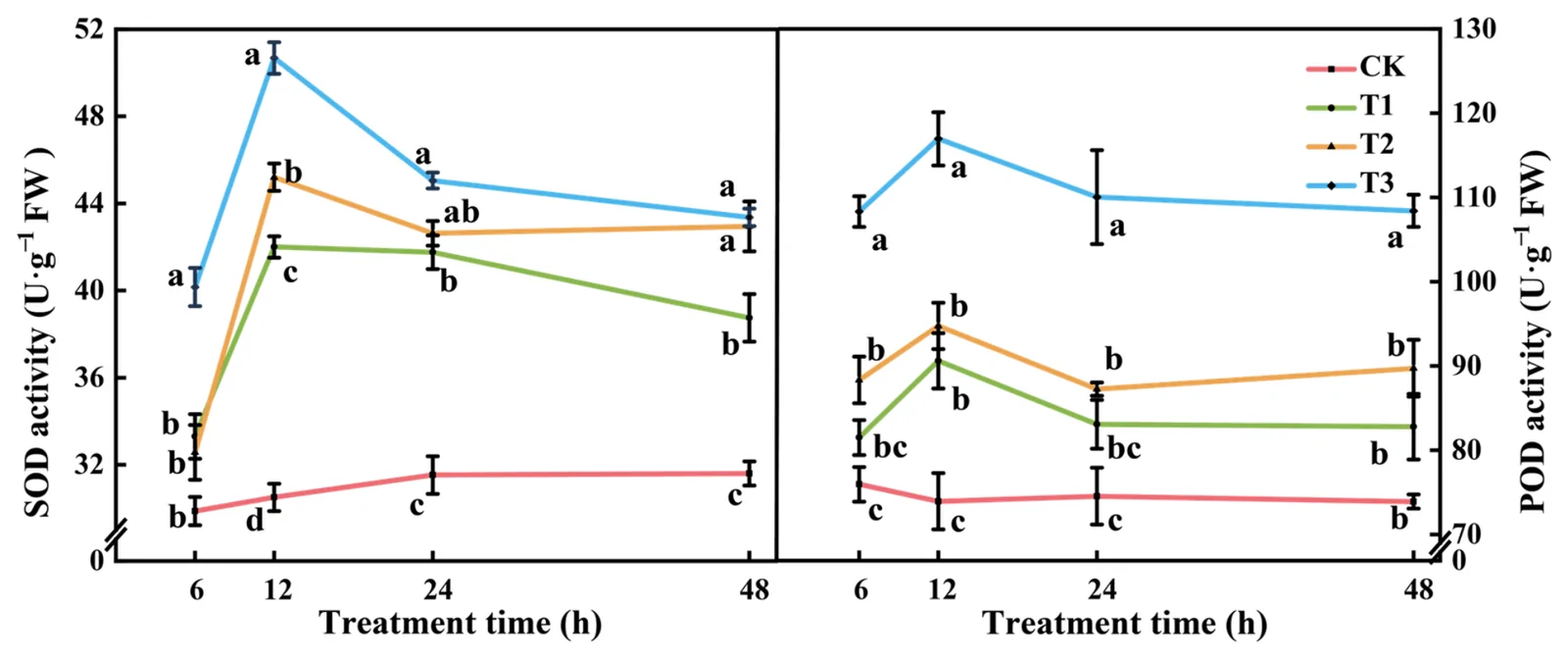
Effects of PEG treatment on SOD and POD activity in apple leaves. Note: CK represents the 0% PEG-6000 treatment, whereas T1, T2, and T3 represent the 5%, 10%, and 15% PEG-6000 treatments, respectively. Data are presented as mean ± standard error (SE). At each sampling time, different letters indicate significant differences among treatments according to Tukey’s honestly significant difference (HSD) test (*p* < 0.05).

At 6–48 h after treatment, both SOD and POD activities in the T1, T2, and T3 treatments significantly exceeded CK levels (Figure 4). The activities of both antioxidant enzymes peaked at 12 h and then declined or tended to stabilize, while remaining significantly higher than those in CK overall. The T3 treatment showed the greatest increases, with SOD and POD activities at 12 h being 37.3% and 46.7% higher than those in CK, respectively. Across the 6, 12, 24, and 48 h sampling times, SOD and POD activities generally tended to increase with increasing PEG-6000 concentration, although significant differences among PEG-treated groups varied among sampling times. The concentration-related differences were most pronounced at 12 h: SOD activity was significantly higher in T2 and T3 than in T1 (*p* = 0.0283 and *p* = 4.64 × 10^−5^), and was also significantly higher in T3 than in T2 (*p* = 0.0012); POD activity in T3 was significantly higher than that in T1 and T2 (*p* = 0.0016 and *p* = 0.0047).

### 3.5. Analysis of Transcriptome Data Quality and Mapping Results in ‘Hanfu’ Apple Leaves

To investigate the transcriptional responses of apple leaves to short-term PEG-simulated drought stress, seedlings subjected to 15% PEG-6000 treatment (T3) for 6 and 24 h were selected for transcriptome analysis based on the changes observed in the physiological parameters. A total of 498,267,772 raw reads were obtained from the sequenced samples. After quality control, a total of 487,903,916 high-quality clean reads were obtained, yielding approximately 72.17 Gb of clean bases. The GC content of all samples was higher than 46.85%, and the per-base error rate of all samples was below 0.03%. The Q20 and Q30 values were at least 97.64% and 90.32%, respectively (Table S2). The mapping rate of clean reads for each sample was at least 85.62%, while the proportion of multi-mapped reads was no more than 10% (Table S3). The results indicated that the RNA-seq data were of high quality and showed good mapping performance, making them suitable for subsequent differential expression and functional enrichment analyses.

### 3.6. Analysis of DEGs

**Figure 5:**
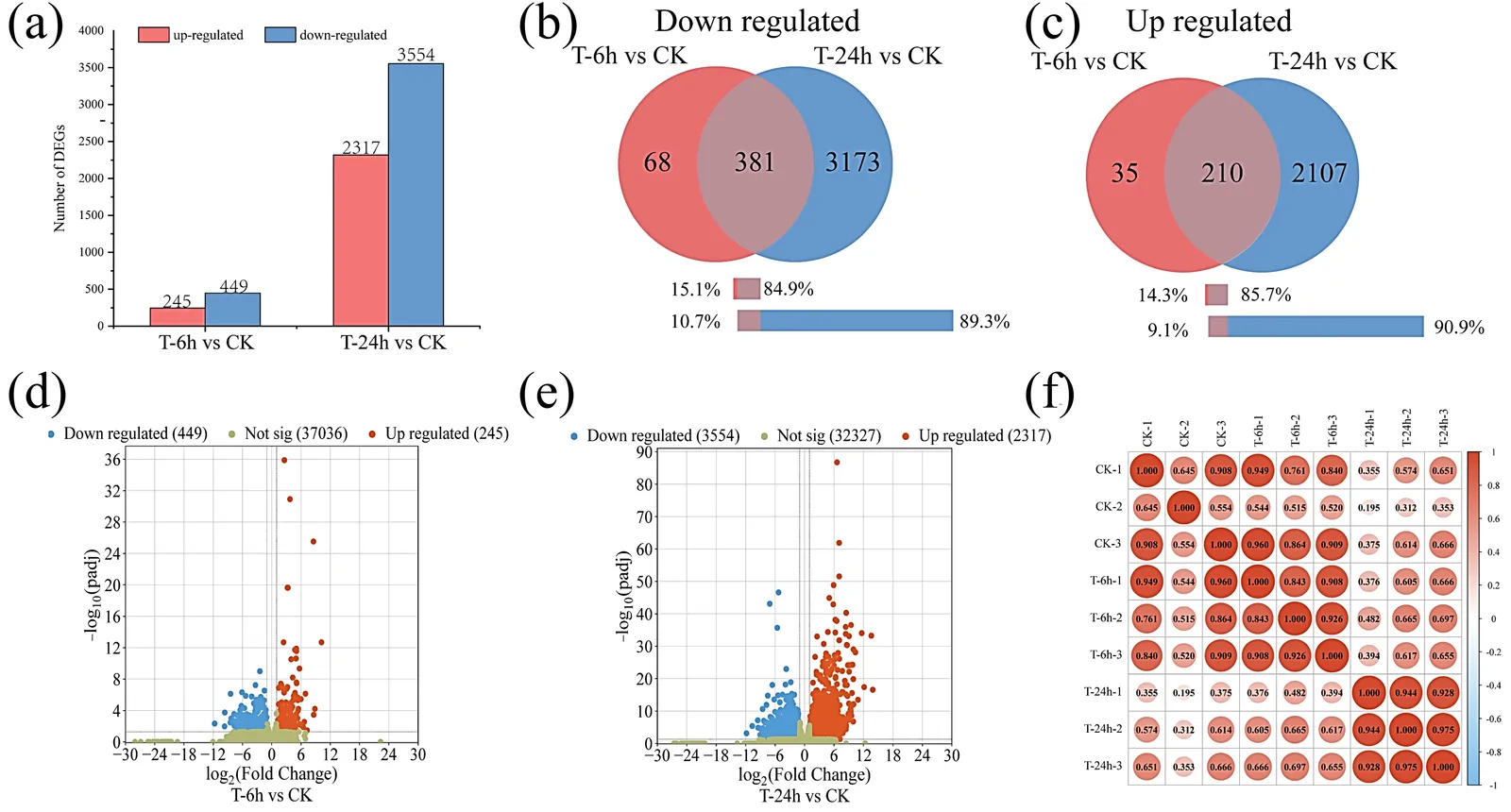
DEGs in the T-6h vs. CK and T-24h vs. CK comparison groups. (**a**) Distribution of DEGs; (**b**) Venn diagram showing downregulated DEGs; (**c**) Venn diagram showing upregulated DEGs; (**d**) volcano plot of the T-6h vs. CK comparison group; (**e**) volcano plot of the T-24h vs. CK comparison group; (**f**) correlation heatmap among samples.

The Pearson correlation coefficients among samples were high, and samples from the same treatment clustered closely, indicating strong consistency among the three biological replicates and high reliability of the transcriptome data (Figure 5f). Differential expression analysis identified 694 DEGs in the T-6h vs. CK comparison, comprising 245 upregulated and 449 downregulated genes. By contrast, 5871 DEGs were detected in the T-24h vs. CK comparison, of which 2317 were upregulated and 3554 were downregulated (Figure 5a). Venn diagram analysis showed that 591 DEGs were common to the T-6h and T-24h comparisons, including 210 commonly upregulated genes and 381 commonly downregulated genes (Figure 5b,c). Volcano plot analysis showed that the number of downregulated genes was greater than that of upregulated genes in both comparisons. Moreover, the T-24h vs. CK comparison showed a wider distribution and a greater number of DEGs than the T-6h vs. CK comparison (Figure 5d,e).

### 3.7. GO Enrichment Analysis of DEGs in Apple Leaves Under Different PEG-Simulated Drought Stress Durations

**Figure 6:**
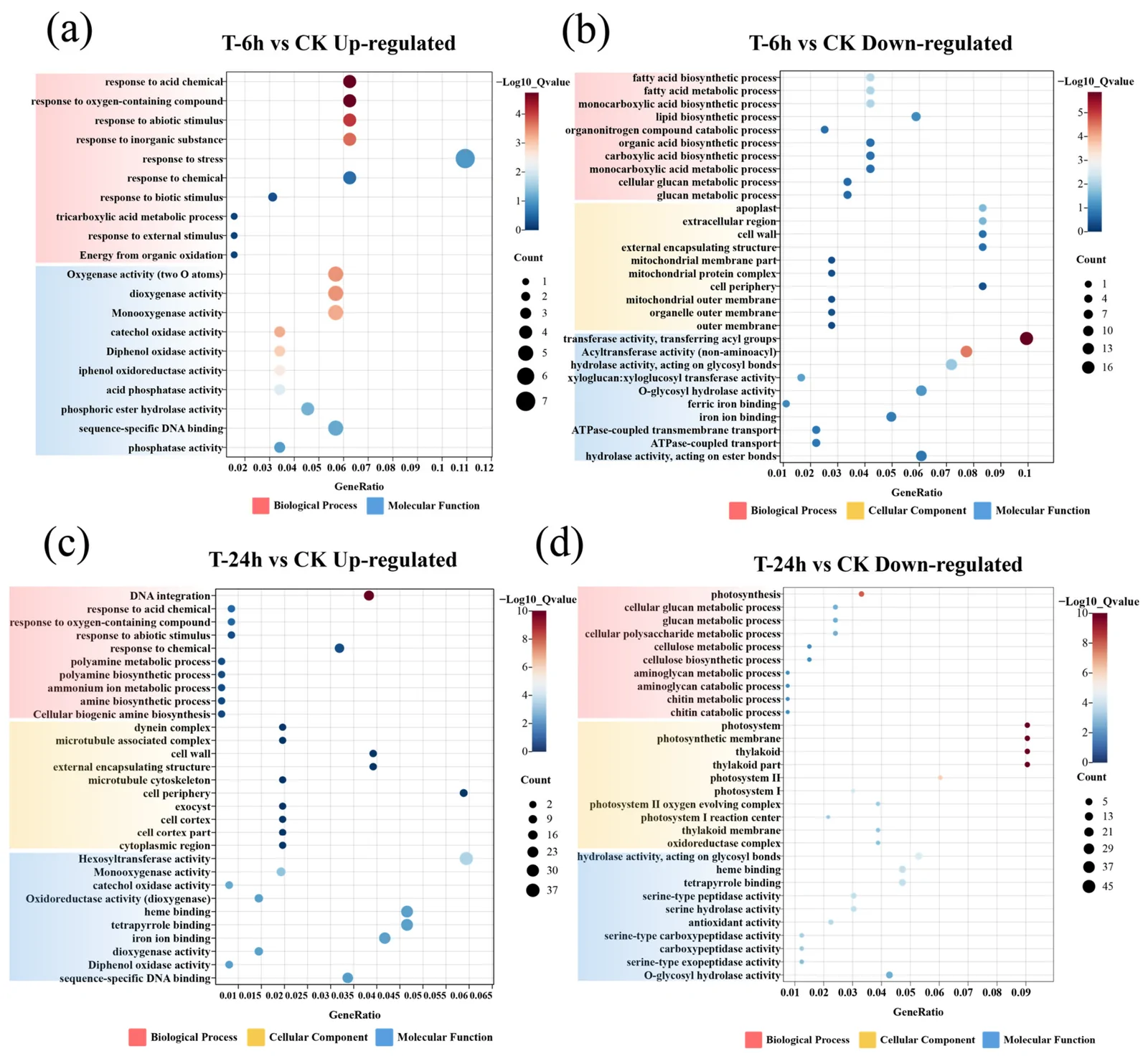
GO enrichment profiles of DEGs in the T-6h vs. CK and T-24h vs. CK comparisons. (**a**) GO terms showing significant enrichment among upregulated DEGs in T-6h vs. CK; (**b**) GO terms showing significant enrichment among downregulated DEGs in T-6h vs. CK; (**c**) GO terms showing significant enrichment among upregulated DEGs in T-24h vs. CK; (**d**) GO terms showing significant enrichment among downregulated DEGs in T-24h vs. CK.

GO enrichment analysis revealed that, in the T-6h vs. CK comparison, upregulated DEGs were mainly associated with BP terms involving responses to stress and chemicals. In the MF category, these DEGs were mainly enriched in oxidoreductase-related terms, including oxidoreductase activity, acting on diphenols and related substances as donors (GO:0016679), and catechol oxidase activity (GO:0004097) (Figure 6a). In contrast, downregulated DEGs were predominantly enriched in lipid metabolism-related BP. The enriched terms included fatty acid biosynthetic process (GO:0006633) and fatty acid metabolic process (GO:0006631). Monocarboxylic acid biosynthetic process (GO:0072330) was also significantly enriched. In CC category, apoplast (GO:0048046) and extracellular region (GO:0005576) represented the principal enriched terms for these DEGs. Within the MF category, downregulated DEGs showed significant enrichment for acyltransferase-related terms, including transferase activity, transferring acyl groups (GO:0016746), and transferase activity, transferring acyl groups other than amino-acyl groups (GO:0016747), as well as iron ion binding (GO:0005506) and several oxidoreductase activity-related terms (Figure 6b).

In the T-24h vs. CK comparison, upregulated DEGs showed prominent enrichment in BP involving carbohydrate metabolism and cell wall polysaccharide metabolism. Within the CC category, cell wall (GO:0005618) and external encapsulating structure (GO:0030312) were the predominant enriched terms, whereas several oxidoreductase-related terms were enriched in the MF category (Figure 6c). In contrast, downregulated DEGs showed predominant enrichment in BP related to photosynthesis and carbohydrate metabolism. The enriched terms included photosynthesis (GO:0015979) and cellular carbohydrate metabolic process (GO:0044262). Glucan metabolic process (GO:0044042) and cellulose biosynthetic process (GO:0030244) were also significantly enriched. In the CC category, these DEGs were mainly associated with photosystem (GO:0009521) and thylakoid-related structures, including thylakoid (GO:0009579). In the MF category, significant enrichment was observed in heme binding (GO:0020037), tetrapyrrole binding (GO:0046906), iron ion binding (GO:0005506), multiple oxidoreductase activity-related terms, peroxidase activity (GO:0004601), antioxidant activity (GO:0016209), multiple glycosyltransferase activity-related terms, and cellulose synthase activity (GO:0016759) (Figure 6d).

### 3.8. KEGG Enrichment Analysis of DEGs in Apple Leaves Under Different Drought Treatment Durations

**Figure 7:**
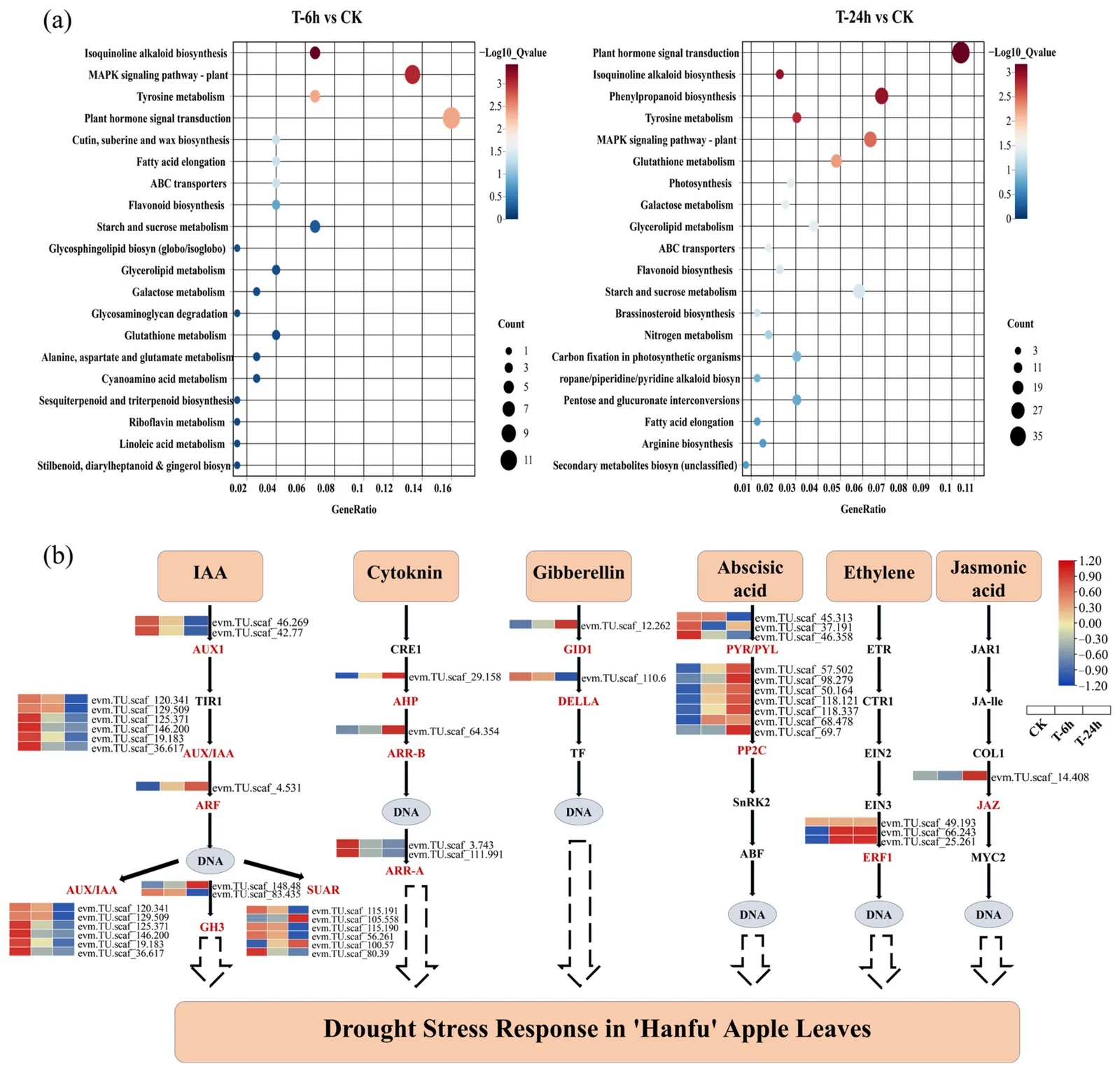
KEGG pathway enrichment and expression patterns of DEGs associated with plant hormone signal transduction under PEG-simulated drought stress. (**a**) Enriched KEGG pathways identified from DEGs in the T-6h vs. CK and T-24h vs. CK comparisons; (**b**) schematic representation of the plant hormone signal transduction pathway together with the expression patterns of the associated DEGs. Solid arrows indicate the direction of signal transduction, whereas dashed arrows indicate downstream connections to the drought stress response.

To further characterize pathway-level responses of apple leaves to PEG-simulated drought stress, KEGG enrichment analysis was conducted using DEGs from the T-6h vs. CK and T-24h vs. CK comparisons (Figure 7a). With FDR ≤ 0.05 as the significance threshold, seven pathways were enriched in T-6h vs. CK: isoquinoline alkaloid biosynthesis (mdm00950), MAPK signaling pathway—plant (mdm04016), tyrosine metabolism (mdm00350), plant hormone signal transduction (mdm04075), cutin, suberine and wax biosynthesis (mdm00073), fatty acid elongation (mdm00062), and ABC transporters (mdm02010). By contrast, 12 pathways were significantly enriched in T-24h vs. CK, namely plant hormone signal transduction (mdm04075), isoquinoline alkaloid biosynthesis (mdm00950), phenylpropanoid biosynthesis (mdm00940), tyrosine metabolism (mdm00350), MAPK signaling pathway—plant (mdm04016), glutathione metabolism (mdm00480), photosynthesis (mdm00195), galactose metabolism (mdm00052), glycerolipid metabolism (mdm00561), ABC transporters (mdm02010), flavonoid biosynthesis (mdm00941), and starch and sucrose metabolism (mdm00500).

Among the enriched pathways, plant hormone signal transduction was significantly enriched in both the T-6h vs. CK and T-24h vs. CK comparisons, indicating that hormone signaling may serve a key regulatory function during drought responses in apple. Further examination of the DEGs within this pathway indicated the involvement of multiple hormone signaling pathways in the drought response of ‘Hanfu’ apple. These included auxin (IAA), cytokinin (CTK), gibberellin (GA), abscisic acid (ABA), ethylene (ETH), and jasmonic acid (JA) signaling pathways (Figure 7b). In the IAA signaling pathway, the major DEGs belonged to *AUX1*, *AUX/IAA*, *ARF*, *GH3*, and *SAUR*, and most of these genes were downregulated under PEG-simulated drought stress. The DEGs associated with the CTK signaling pathway mainly included *AHP*, *ARR-B*, and *ARR-A*. Genes related to *AHP* and *ARR-B* were upregulated, whereas ARR-A-related genes remained downregulated throughout the drought treatment. In the GA signaling pathway, the principal DEGs included *GID1* and *DELLA*. GID1-related genes showed sustained upregulation, whereas DELLA-related genes exhibited sustained downregulation under PEG-simulated drought stress. The ABA signaling pathway mainly contained DEGs associated with *PYR/PYL* and *PP2C*. Most *PYR/PYL* genes were downregulated after drought treatment, although the expression of some members increased again at 24 h, while most *PP2C* genes were upregulated. In the ETH signaling pathway, *ERF1* was among the major DEGs and showed a clear upregulation trend following drought treatment. In contrast, the DEGs identified for JA signaling were predominantly associated with *JAZ*, which exhibited a sustained downregulation trend under PEG-simulated drought stress.

### 3.9. Analysis of Differentially Expressed Transcription Factors in Apple Leaves Under Different Drought Treatment Durations

**Figure 8:**
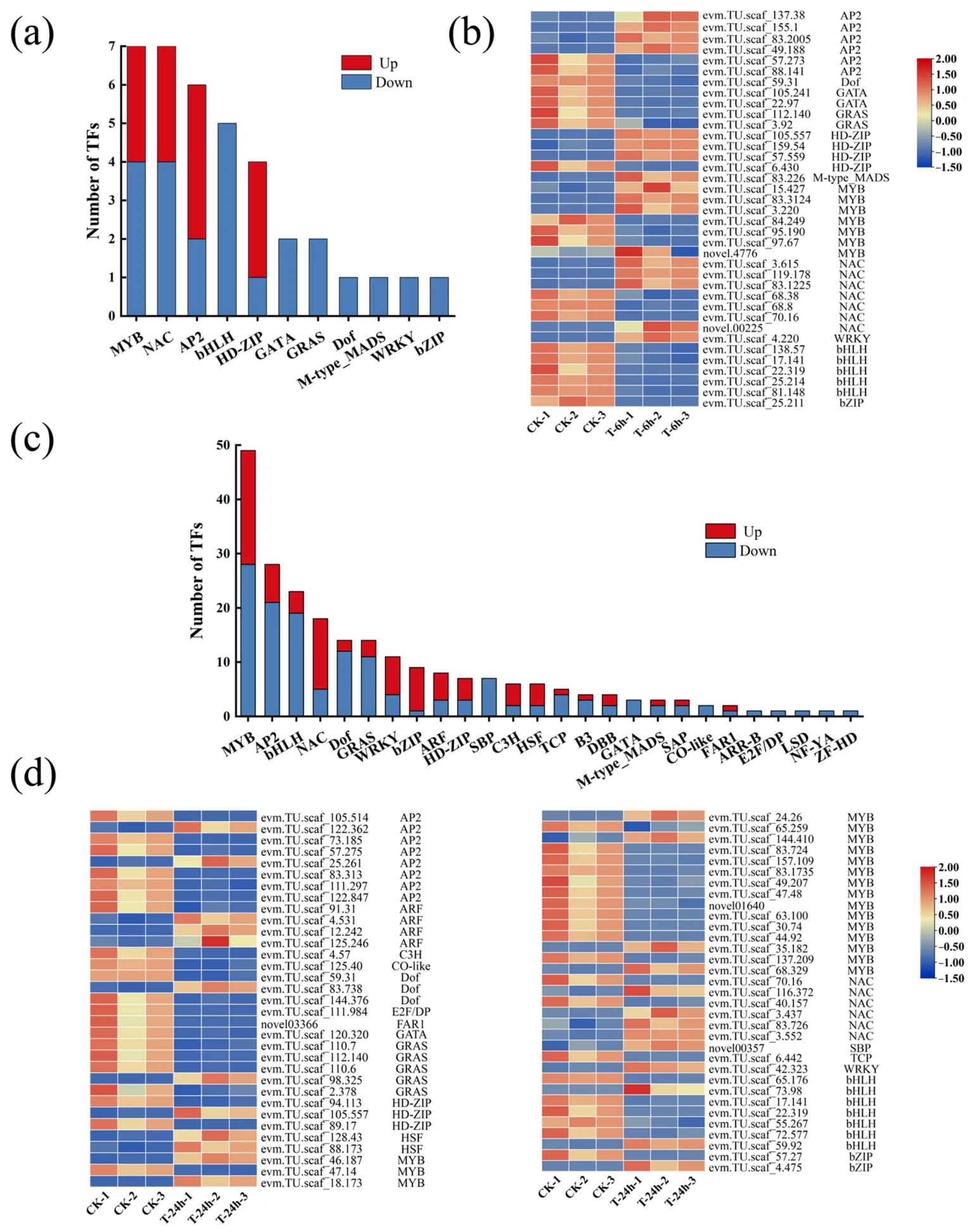
Distribution and expression profiles of differentially expressed transcription factors under PEG-simulated drought stress. (**a**) Numbers of up- and downregulated transcription factors in the T-6h vs. CK comparison; (**b**) heatmap of differentially expressed transcription factors in T-6h vs. CK; (**c**) numbers of up- and downregulated transcription factors in the T-24h vs. CK comparison; (**d**) heatmap of differentially expressed transcription factors in T-24h vs. CK.

As shown in Figure 8a,b, 13 upregulated and 24 downregulated transcription factor genes were identified in the T-6h vs. CK comparison. Most differentially expressed *AP2* and *HD-ZIP* transcription factor genes were upregulated, whereas *bHLH*, *GATA*, *Dof*, *M-type*, *MADS*, *WRKY*, and *bZIP* transcription factor genes were downregulated. As shown in Figure 8c,d, 90 upregulated and 141 downregulated transcription factors were identified in the T-24h vs. CK comparison. Most differentially expressed transcription factor genes were downregulated. Among these, the *MYB* family contained the largest number of genes and showed a pronounced response at both treatment time points.

### 3.10. qRT-PCR Validation

**Figure 9:**
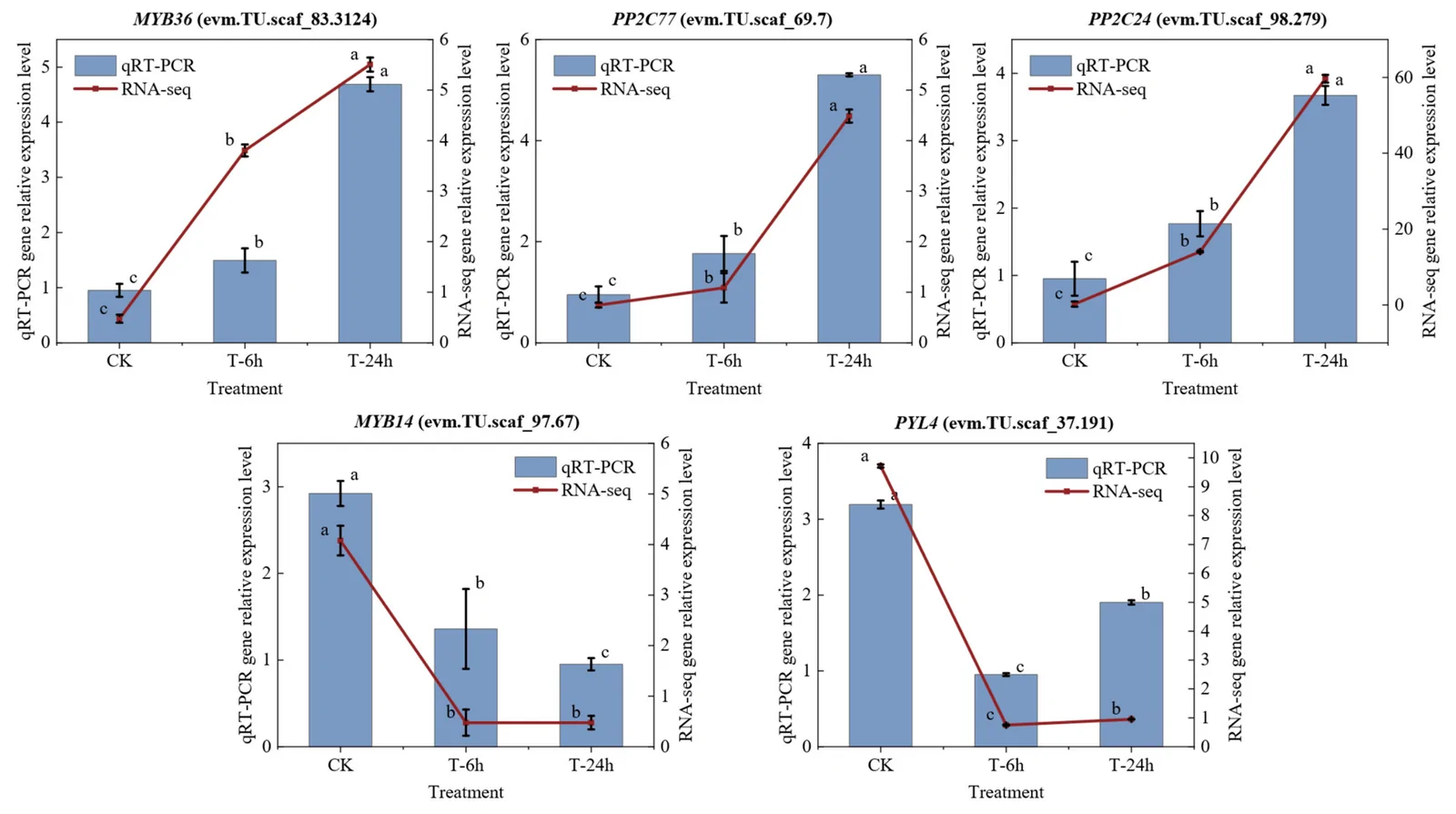
qRT-PCR validation of selected DEGs. Different lowercase letters indicate significant differences among treatments within each dataset (*p* < 0.05, Tukey’s HSD test).

Five genes showing significant differential expression at both 6 and 24 h were chosen for qRT-PCR validation: the ABA receptor gene *PYL4*, the protein phosphatase genes *PP2C77* and *PP2C24*, and the transcription factor genes *MYB14* and *MYB36*. As shown in Figure 9, all five genes showed expression trends in agreement with the RNA-seq results, supporting the reliability of the transcriptomic data and providing a basis for further investigation of the identified DEGs.

## 4. Discussion

Physiological measurements were integrated with transcriptome analysis in this study. This approach characterized drought-induced physiological alterations and molecular regulatory patterns in leaves of ‘Hanfu’ apple, a cultivar reported to possess drought resistance [18]. Physiological measurements showed that, with increasing duration and concentration of PEG-6000 treatment, leaf water potential and the contents of chlorophyll a, total chlorophyll, and carotenoids gradually decreased. These findings suggest that short-term PEG-simulated drought stress exacerbated water deficit in apple leaves and affected photosynthetic pigment metabolism and photosynthesis-related processes [4]. Chlorophyll b content increased transiently during the early stage of stress, possibly reflecting an adaptive adjustment of the light-harvesting system in apple leaves [29]. As the stress persisted, this compensatory response gradually weakened, resulting in an overall decline in photosynthetic pigment contents and the inhibition of photosynthesis [30]. At 6 h, significant enrichment of the photosynthesis-antenna protein pathway was observed in the transcriptome analysis, whereas the photosynthesis pathway showed significant enrichment at 24 h. This temporal difference suggests that the photosynthetic response to PEG-simulated drought stress developed progressively from early regulation of light harvesting to broader changes in photosystem function and electron transport. The photosynthesis-related genes identified at each time point were all downregulated, although they were associated with different functional processes. At 6 h, *CAB40*, *CAB21*, *lhcA-P4*, *CAB6A*, *CAB13*, and a candidate gene encoding the CP24 protein were downregulated and were mainly associated with light harvesting and antenna protein function. The expression of *NtCAB-40* and *NtCAB-21* is closely associated with chlorophyll accumulation, photosynthetic activity, and photosynthetic efficiency, while silencing of *Lhc* genes in tomato resulted in reduced chlorophyll content and inhibited photosynthesis [31,32]. Similarly, in the drought-tolerant *Zanthoxylum bungeanum* cultivar ‘Hanjiao’, several light-harvesting-related genes, including *LhcB4.3*, *LhcA4*, *LhcB4.2*, *CAB-151*, and *CAB8*, were also downregulated under drought stress [30]. These findings support the view that regulation of the light-harvesting system is an important component of the early photosynthetic response to water-deficit-related stress. At 24 h, *PNSL2*, *PNSL3*, *PSAL*, *PSAG*, *PSAH2*, and *PSAD* were downregulated and were mainly associated with the NDH complex and photosystem I (PSI) function. Among them, *PNSL2* and *PNSL3* encode thylakoid lumen proteins associated with the chloroplast NAD(P)H dehydrogenase-like (NDH) complex and contribute to NDH–PSI supercomplex assembly and cyclic electron flow around PSI [33,34].Whereas PSAL and PSAH participate in state transitions and contribute to the redistribution of LHCII-derived excitation energy toward PSI [35]. Similarly, in the drought-tolerant soybean cultivar HN44, the PSI-related genes *PSAH* and *PSAG* were downregulated as water deficit intensified [36]. Based on these findings, at 24 h, the coordinated downregulation of these PSI- and NDH-related genes may compromise PSI structural stability, impair cyclic electron flow, and disrupt energy distribution between the photosystems, thereby further exacerbating the decline in leaf photosynthetic function under PEG-simulated drought stress.

In this study, exposure to different concentrations of PEG-6000 significantly increased O_2_·^−^, H_2_O_2_, and MDA levels in apple leaves, indicating that PEG-simulated drought stress induced oxidative damage. POD and SOD activities generally exceeded CK levels and initially increased before declining, indicating that apple leaves enhanced their ROS-scavenging capacity by increasing antioxidant enzyme activities at the early stage of PEG-simulated drought stress. As PEG-simulated drought stress progressed (24–48 h), ROS and MDA levels and antioxidant enzyme activities gradually stabilized under some treatments, suggesting that a new dynamic balance may have been established between oxidative stress and antioxidant defense in the leaves. Relatively high antioxidant enzyme activities can facilitate the scavenging of excess ROS and inhibit membrane lipid peroxidation, thereby alleviating drought-induced oxidative damage in ‘Hanfu’ apple [5]. Transcriptome analysis further showed that redox-related transcriptional responses occurred at both 6 h and 24 h, although the specific processes involved differed between the two time points. At 6 h, DEGs were predominantly enriched in oxidation-reduction-related processes and the tyrosine metabolism pathway. These results indicate that redox regulation and phenolic compound-related metabolic processes were activated at an early point in the drought response [37]. Together with the enhanced SOD and POD activities observed during the early treatment period, these transcriptional changes suggest that redox regulation and enzymatic antioxidant defense were rapidly mobilized following PEG-induced osmotic stress. At this time point, PPO-related genes were upregulated. In *Prunus mira*, Drought stress strongly upregulated *PmPPO*, while its overexpression enhanced antioxidant enzyme activities and ROS-scavenging capacity, alleviated cellular damage, and enhanced drought tolerance [38]. Therefore, the early upregulation of *PPO*-related genes observed in the present study may contribute to the adaptation of ‘Hanfu’ apple leaves to short-term PEG-simulated drought stress by regulating ROS homeostasis. At 24 h, glutathione metabolism, phenylpropanoid biosynthesis, and flavonoid biosynthesis, which are involved in ROS scavenging and antioxidant defense, were significantly enriched [39]. Compared with the early redox response observed at 6 h, these changes indicate that the antioxidant response had expanded by 24 h to involve a broader network of antioxidant and secondary metabolic processes. Among them, *APX3*, *PK1*, *APX2*, *PER40*, and *PER9* were upregulated. A functional study in maize showed that overexpression of *ZmAPX2* reduced ROS accumulation and MDA content, increased APX, POD, and SOD activities, thereby increasing drought tolerance [40]. In addition, *PER40* and *PER9* have been associated with antioxidant regulation under drought stress, suggesting that class III peroxidase-mediated redox regulation may play a role in plant drought responses [41,42]. The coordinated upregulation of *APX2*, *APX3*, *PER40*, *PER9*, and *PK1* suggests that adjustments in antioxidant defense and energy metabolism may form part of the drought response of ‘Hanfu’ apple leaves.

Plant hormone signaling is crucial for drought perception and coordinates downstream stress responses [43]. Rapid hormone-signaling responses have also been reported during the early stage of osmotic stress in apple. Hou et al. found that several *MdPYL* genes responded strongly after 6 h of PEG treatment and reached their highest expression levels at 12 h, indicating that ABA receptor-related signaling can be activated within the first several hours of osmotic stress [44]. In this study, numerous DEGs mapped to the plant hormone signal transduction pathway. These DEGs were distributed across IAA, CTK, GA, ABA, ETH, and JA signaling. Among these pathways, the IAA and ABA signaling pathways contained a greater number of DEGs. This indicates that these two pathways may contribute substantially to the short-term drought response of ‘Hanfu’ apple leaves. In the IAA signaling pathway, *LAX1* and *LAX2* were downregulated, suggesting that PEG-simulated drought stress may affect auxin transport and distribution. Similarly, transcriptomic analysis of maize kernels under drought stress showed that several AUX1/LAX auxin influx carrier genes, including those annotated as *LAX1* and *LAX2*, were markedly downregulated, accompanied by reduced polar auxin transport [45]. In this study, *GH3.5* was downregulated, whereas *GH3.1* was upregulated. Functional analysis in apple showed that overexpression of *MdGH3.6* reduced IAA content and drought tolerance [46]. *SAUR20* was downregulated, whereas two genes annotated as *SAUR32* showed contrasting expression patterns, with one upregulated and the other downregulated. In *Arabidopsis*, overexpression of *AtSAUR32* enhanced drought tolerance, whereas the *saur32* mutant exhibited higher electrolyte leakage and lower ABA accumulation under drought stress [47]. Collectively, these expression patterns suggest that IAA signaling is regulated in a gene-specific manner under short-term PEG-simulated drought stress and may interact with ABA signaling.

In the ABA signaling pathway, *PYL4* was downregulated, whereas multiple *PP2C* genes were upregulated, indicating marked transcriptional regulation of the core PYL–PP2C signaling module. Although several *MdPYL* genes were rapidly upregulated after PEG treatment in a previous apple study [44], *PYL4* and several other PYR/PYL-related genes showed predominantly decreased expression in the present study. This difference suggests that the early transcriptional regulation of ABA receptor genes may vary among individual PYL family members, genotypes, tissues, and treatment conditions. Nevertheless, both studies indicate that the PYL–PP2C module responds during the early stage of osmotic stress. A functional study of the tomato ABA receptor gene *SlPYL4* showed that silencing of *SlPYL4* resulted in more severe wilting and greater H_2_O_2_ and O_2_·^−^ accumulation under drought stress, accompanied by reduced drought tolerance [48]. Furthermore, a comparative transcriptomic study involving drought-tolerant *Malus prunifolia* and drought-sensitive *Malus hupehensis* revealed that ABA biosynthesis and signaling were activated more rapidly in *M. prunifolia* under mild and moderate drought stress, with PYL-related components involved in the early ABA response [49]. These findings indicate that PYL- and PP2C-related components are important elements of ABA-dependent drought responses in apple. Therefore, the downregulation of *PYL4* and upregulation of multiple *PP2C* genes detected in the present study may reflect dynamic regulation of the core ABA signaling module in ‘Hanfu’ apple leaves under short-term PEG-simulated drought stress. In addition to IAA and ABA signaling, genes associated with CTK, GA, ETH, and JA signaling were also differentially expressed, suggesting that the short-term drought response of ‘Hanfu’ apple leaves may involve multiple hormone signaling pathways. Previous studies have indicated that CTK, GA, ETH, and JA signaling are involved in plant responses to drought stress and may exhibit crosstalk with ABA signaling in regulating plant growth and stress adaptation [50,51,52,53]. Previous studies have reported that ‘Hanfu’ exhibits drought tolerance [18]. The differential expression of genes associated with these hormone signaling pathways may be related to the adaptation of ‘Hanfu’ apple leaves to short-term PEG-simulated drought stress.

Transcriptome analysis revealed that, compared with the T-6h vs. CK comparison, the T-24h vs. CK comparison contained more DEGs and differentially expressed transcription factors, which also represented a broader range of families. These findings indicate that the transcriptional response of ‘Hanfu’ apple leaves became more pronounced as drought stress progressed from 6 to 24 h [54]. The marked increase in the number of DEGs at 24 h suggests a transition from a relatively selective early response to broader transcriptional reprogramming as the stress progressed. This expansion likely reflects the recruitment of additional regulatory and metabolic processes, including photosynthesis, carbohydrate metabolism, antioxidant metabolism, hormone signaling, and transcriptional regulation, to coordinate continued adjustment to PEG-simulated drought stress. Meanwhile, at 24 h, repression affected a broader set of genes involved in plant growth, development, and primary metabolism [55]. Transcription factors play central roles in regulating plant responses to environmental stress. Numerous transcription factor families, including MYB, NAC, WRKY, and AP2/ERF, participate in responses to abiotic stresses such as drought and salinity by modulating genes involved in hormone signaling, antioxidant defense, and secondary metabolism, thereby influencing plant stress tolerance [56,57]. Rapid transcription factor responses have also been observed during the first several hours of osmotic stress in apple. In ‘GL-3’ apple seedlings, *MdNAC29* was rapidly induced by PEG treatment and reached its maximum expression level at 3 h, while the drought-responsive gene *MdDREB2A* reached its highest expression level at 1 h [58]. In addition, *MsMYB88* and *MsMYB124* in *Malus sieversii* responded to simulated drought treatment within 6 h [11]. Significant drought-responsive expression changes were observed in transcription factors from the MYB, WRKY, bZIP, AP2, bHLH, Dof, NAC, and ARF families. Notably, most differentially expressed AP2 and HD-ZIP transcription factor genes were upregulated at 6 h, suggesting that these families may participate in the early transcriptional response to PEG-induced osmotic stress. AP2/ERF transcription factors in apple have been shown to respond to osmotic and other abiotic stresses, supporting their involvement in early stress signaling and downstream gene regulation [59]. In addition, the apple HD-ZIP I transcription factor *MdHB-7* positively regulates adaptation to water deficit by affecting stomatal density, photosynthetic performance, root activity, and water-use efficiency [60]. Therefore, the early upregulation of *AP2* and *HD-ZIP* genes observed in the present study may reflect the rapid activation of transcriptional regulatory mechanisms associated with stress perception and water-deficit adjustment, although the functions of the individual genes identified here require further validation. Transcriptomic comparison between drought-tolerant *Malus prunifolia* and drought-sensitive *Malus hupehensis* showed that NAC, WRKY, and MYB transcription factors were induced more rapidly in *M. prunifolia* under mild and moderate drought stress [49]. In the present study, MYB, WRKY, and NAC transcription factors also showed marked responses, consistent with previous findings that these transcription factor families are involved in apple drought responses, suggesting that transcription factor-mediated regulation may be an important component of the short-term drought response of ‘Hanfu’ apple leaves.

Although previous studies have reported that ‘Hanfu’ exhibits drought tolerance [18]. The present study included only a single apple cultivar and did not include genotypes with contrasting drought tolerance. In addition, the PEG-6000 root-immersion system used in this study primarily imposed osmotic stress by reducing external water potential and did not fully reproduce the complex changes associated with soil drought, such as changes in root-zone oxygen availability, soil mechanical resistance, temperature, nutrient availability, and root architecture. Another limitation of the present study is the absence of a 0 h measurement before PEG-6000 treatment. The original experimental design focused on comparing the responses of PEG-treated seedlings with those of the corresponding CK plants at 6, 12, 24, and 48 h. Therefore, although treatment-related differences could be evaluated at the selected sampling times, the lack of an initial baseline limits the ability to determine the magnitude of physiological and transcriptional changes relative to the pre-treatment state. Consequently, the physiological and transcriptional changes observed in this study should be interpreted as responses to PEG-simulated drought stress rather than as evidence of drought-tolerance mechanisms or as responses fully equivalent to actual soil drought, and the temporal patterns should be interpreted primarily as differences among treatments and sampling times rather than as absolute changes from the initial physiological state. The roles of the candidate genes require further comparative analysis and functional validation, preferably using genotypes with contrasting drought tolerance, soil-based drought conditions, and an initial 0 h baseline.

## 5. Conclusions

Our findings revealed that PEG-simulated drought stress exacerbated water deficit and reduced photosynthetic pigment contents in ‘Hanfu’ apple leaves. Photosynthesis-related genes, including *CAB*, *CP24*, *PSA*, and *PNSL2*, were significantly downregulated. The contents of ROS and MDA increased, and antioxidant enzyme activities were enhanced. Antioxidant-related genes, including *PPO*, *APX*, *PK1*, and *PER*, were significantly upregulated. Transcriptome analysis also revealed marked differential expression of genes including plant hormone signal transduction. In the IAA signaling pathway, *LAX*, *IAA*, *GH3.5*, *SAUR*, and *AX6B* were significantly downregulated, whereas *ARF5*, *GH3.1*, *SAUR32*, and *ARG7* were significantly upregulated. Within the ABA signaling pathway, *PYL4* showed significant downregulation. In contrast, multiple genes, including *PP2C*, *ABI*, and *HAB1*, were upregulated under PEG-simulated drought stress. MYB, WRKY, bZIP, AP2, bHLH, Dof, NAC, and ARF may be the major transcription factor families associated with drought-stress responses.

## Data Availability

The original contributions presented in this study are included in the article. Further inquiries can be directed to the corresponding authors.

## Supplementary Materials

The following supporting information can be downloaded at: https://www.mdpi.com/article/10.3390/biology15201861/s1, Table S1: qPT-PCR primer sequence; Table S2: Summary of sequences analysis in apple leaves; Table S3: BLAST analysis of unigenes against reference reads.
