# Supplementary material for: Physiological Responses and Transcriptome Analysis of ‘Hanfu’ Apple Plants Under PEG-Simulated Drought Stress
Source: Biology (Basel). 2026 Oct 16;15(20):1861. doi: 10.3390/biology15201861 (PMC13701644; doi:10.3390/biology15201861)
Supplement: Supplementary file 1 [file biology-15-01861-s001.zip › biology-4498987-supplementary.pdf]

Supplementary Materials

Supplementary Table:

Table S1. qPT-PCR primer sequence

Gene names	Primer-Forward（5′-3′）	Primer-Reverse（5′-3′）
Apple-Actin	TACTCAGCTTTGGCAATCCACATC	TGACCGAATGAGCAAGGAAATTACT
MYB 36	TGGTCTGCTATTGCTGCTCG	AAGTTGTTCATTTGATGGTGGTG
MYB 14	GTGATGGCGGGGTCTGTC	AGGAGTTGTTGCATAGTCATTCG
PP2C 24	GCTGGTGAAGGAGGAAGTGG	GAAGCTCGCAACGGCAAT
PP2C 77	GCTCAGGCAGTTGCTGTTCC	CGACTTCCACTCCCCGTTT
PYL 4	GACCATCGGCTGACGAACTA	TTCTTGGTGTTTGGCTTATCG

Table S2. Summary of sequences analysis in apple leaves.

**Sample**	**Raw Reads**	**Clean reads**	**Error（%）**	**Q20（%）**	**Q30（%）**	**GC content（%）**
CK-0h-1	56551670	55765992	0.03	97.80	93.74	47.29
CK-0h-2	63476580	62495076	0.03	97.79	93.70	46.99
CK-0h-3	52584046	51258588	0.03	97.66	93.41	46.69
T-6h-1	54624528	53054810	0.03	97.86	93.86	46.87
T-6h-2	54077702	52735586	0.03	97.79	93.68	46.53
T-6h-3	56790426	55589402	0.03	97.85	93.81	46.48
T-24h-1	48749844	47944806	0.03	97.77	93.63	47.07
T-24h-2	50242364	49077578	0.03	97.7	93.48	46.79
T-24h-3	54245702	53252994	0.03	97.64	93.32	46.91
Total	498267772	487903916				

Note: Q20: The percentage of bases with a Phred value > 20; Q30: The percentage of bases with a Phred value > 30. CK-0h、T-6h、T-24h means: Control , severe drought was treated at 6h and severe drought was treated at 24h.

Table S3. BLAST analysis of unigenes against reference reads.

**Sample name**	**Clean reads**	**Total mapped**	**Multiple mapped**
CK_0h_1	55765992	48180250 (86.4%)	4658993 (8.35%)
CK_0h_2	62495076	48180250 (86.4%)	4658993 (8.35%)
CK_0h_3	51258588	48180250 (86.4%)	4658993 (8.35%)
T_6h_1	53054810	45424099 (85.62%)	4478437 (8.44%)
T_6h_2	52735586	45424099 (85.62%)	4478437 (8.44%)
T_6h_3	55589402	45424099 (85.62%)	4478437 (8.44%)
T_24h_1	47944806	41170023 (85.87%)	4545496 (9.48%)
T_24h_2	49077578	41170023 (85.87%)	4545496 (9.48%)
T_24h_3	53252994	41170023 (85.87%)	4545496 (9.48%)
